# Breath analysis for the detection of digestive tract malignancies: systematic review

**DOI:** 10.1093/bjsopen/zrab013

**Published:** 2021-04-15

**Authors:** K F H Hintzen, J Grote, A G W E Wintjens, T Lubbers, M M M Eussen, F J van Schooten, N D Bouvy, A Peeters

**Affiliations:** Department of Surgery, Maastricht University Medical Centre, Maastricht, the Netherlands; Department of Pharmacology and Toxicology, Maastricht University, Maastricht, the Netherlands; Department of Surgery, Maastricht University Medical Centre, Maastricht, the Netherlands; Department of Surgery, Maastricht University Medical Centre, Maastricht, the Netherlands; Department of Surgery, Maastricht University Medical Centre, Maastricht, the Netherlands; Department of Surgery, Maastricht University Medical Centre, Maastricht, the Netherlands; Department of Pharmacology and Toxicology, Maastricht University, Maastricht, the Netherlands; Department of Surgery, Maastricht University Medical Centre, Maastricht, the Netherlands; Department of Clinical Epidemiology and Medical Technology Assessment, Maastricht University Medical Centre, Maastricht, the Netherlands

## Abstract

**Background:**

In recent decades there has been growing interest in the use of volatile organic compounds (VOCs) in exhaled breath as biomarkers for the diagnosis of multiple variants of cancer. This review aimed to evaluate the diagnostic accuracy and current status of VOC analysis in exhaled breath for the detection of cancer in the digestive tract.

**Methods:**

PubMed and the Cochrane Library database were searched for VOC analysis studies, in which exhaled air was used to detect gastro-oesophageal, liver, pancreatic, and intestinal cancer in humans, Quality assessment was performed using the QUADAS-2 criteria. Data on diagnostic performance, VOCs with discriminative power, and methodological information were extracted from the included articles.

**Results:**

Twenty-three articles were included (gastro-oesophageal cancer n = 14, liver cancer n = 1, pancreatic cancer n = 2, colorectal cancer n = 6). Methodological issues included different modalities of patient preparation and sampling and platform used. The sensitivity and specificity of VOC analysis ranged from 66.7 to 100 per cent and from 48.1 to 97.9 per cent respectively. Owing to heterogeneity of the studies, no pooling of the results could be performed. Of the VOCs found, 32 were identified in more than one study. Nineteen were reported as cancer type-specific, whereas 13 were found in different cancer types. Overall, decanal, nonanal, and acetone were the most frequently identified.

**Conclusion:**

The literature on VOC analysis has documented a lack of standardization in study designs. Heterogeneity between the studies and insufficient validation of the results make interpretation of the outcomes challenging. To reach clinical applicability, future studies on breath analysis should provide an accurate description of the methodology and validate their findings.

## Teaser

This review provides an overview of the diagnostic performance and current status of volatile organic compound (VOC) analysis in exhaled breath for the detection of cancer in the digestive tract, including gastro-oesophageal, liver, pancreatic, and colorectal cancers. Some VOCs were cancer type-specific, whereas others were found in different cancer types. Their use in cancer screening is discussed.

Breath analysis and gastrointestinal cancer screening

## Introduction

Cancer is one of the leading causes of premature death. With an increasing worldwide life expectancy, the prevalence of cancer and its burden on society is growing^1^. Early-stage cancers are often asymptomatic and therefore difficult to detect. Treatment options for cancer, and ultimately their success rates, are greatly dependent on the disease stage at the time of diagnosis. The 5-year survival rate of stage I colorectal cancer is approximately 97.7 per cent, but it drops to 43.9 per cent for stage IV. Similar reductions in 5-year survival rate are seen in other cancer types, including gastro-oesophageal, liver, and pancreatic cancers^2^. These survival rates indicate the importance of screening programmes in detecting cancers in an early stage. Current screening and diagnostic techniques are often invasive and not patient friendly. This review focuses on the detection of digestive tract malignancies, including colorectal, gastro-oesophageal, liver, and pancreatic cancers, by analysis of exhaled air.

Colorectal cancer is one of the largest causes of cancer-related deaths. Screening for colorectal cancer with known tumour markers, such as carcinoembryonic antigen or cancer antigen 19.9, are not ideal owing to low sensitivity and specificity^3^^,^^4^. Faecal blood tests, such as the guaiac faecal occult blood test and the more recent faecal immunohistochemical test (FIT), can be used as screening tools for colorectal cancer^5^. In 2014, a national screening programme was introduced in the Netherlands using the FIT, leading to earlier diagnosis of colorectal cancer. In the event of a positive test, which indicates an increased risk of colorectal cancer, colonoscopy is recommended^6^. Although the FIT is non-invasive and has a sensitivity of over 80 per cent, a malignancy is found during colonoscopy after only about 8 per cent of positive tests^7^.

Next to colorectal cancer, gastric carcinoma is a common digestive tract malignancy with reported late diagnosis and high mortality rates^8^. In high-incidence countries, including Japan, screening programmes using gastroscopy have shown a decrease in mortality^9^. However, a major drawback of this screening programme is the invasive character of the endoscopic procedures used and the risk of complications.

Liver cancer is far less common. Screening using regular ultrasound imaging is performed in patients with underlying risk factors, such as chronic viral hepatitis or alcohol intake^10^. Although it is non-invasive, its sensitivity is relatively low and is operator-dependent^11^.

The same holds for pancreatic cancer. Pancreatic tumours in less than 20 per cent of patients are operable at the time of diagnosis^12^, and screening (using endoscopic ultrasonography or MRI) is currently recommended only for patients with a genetic predisposition^13^. However, the prognosis is poor, symptoms are associated with disease progression, and deaths from the disease are increasing globally^1^^,^^14^^,^^15^.

There is a general need for improvement in screening techniques for digestive tract malignancies. The sensitivity and specificity of most screening tools are not high enough to reach clinically valuable post-test probabilities in a screening setting. Thus, it remains a challenge within global healthcare to develop more suitable diagnostic tools for tumour detection^16^^,^^17^.

In recent years, detection of cancer by analysis of volatile organic compounds (VOCs) in body materials has shown promising results. VOC analysis has a long-standing history in medical research. By 1971, the Nobel Prize winner Linus Pauling^18^ had detected 250 different compounds in breath using gas chromatography. Today, the value of VOC analysis in exhaled breath has been examined as monitoring tool in many diseases^19^, including the heart transplant rejection breath test^20^.

VOCs are carbon-based organic molecules, and their presence in exhaled breath can be divided into exogenously or endogenously derived compounds, according to their origin. Exogenous VOCs originate from environmental factors, such as food and beverage consumption, smoking, or other environmental exposures. Endogenous VOCs are produced as by- or end-products of human or microbial metabolism. Apart from breath, VOCs can be detected in sweat, blood, tissue samples, urine, and faeces^21^^,^^22^. At present, more than 800 different breath VOCs have been registered in the Chemical Abstracts Service system^22^. The composition of VOCs in exhaled breath can be altered owing to pathological processes such as the presence of cancer. Tumour-associated inflammation leading to enhanced oxidative stress, altered glucose metabolism, and redox regulation in cancer cells can lead to different VOC signatures in patients with cancer^23–25^. Breath analysis methods might be able to identify ‘breath signatures’ specific to those with cancer. This could be of value in clinical practice.

Analysis of VOC profiles can be performed using a variety of analytical platforms^26^. Currently, the most common systems in use are gas chromatography mass spectrometry (GC-MS), proton transfer reaction mass spectrometry (PTR-MS), and selected ion flow tube mass spectrometry (SIFT-MS). In addition, pattern recognition sensor systems are emerging that detect total VOC-binding patterns instead of individual VOCs. The latter systems are commonly referred to as an electronic nose or E-nose^26^^,^^27^. All systems have their strengths and limitations. Systems that allow selective quantification of VOCs are usually more laborious, require trained personnel, and are expensive in comparison to systems that register unselective VOC binding patterns, such as portable E-nose systems^17^.

The non-invasive nature of breath analysis makes it interesting for clinical use. Despite a long history of breath research, there are currently only a few applications in the clinic. This review provides an overview of the current literature on the identification of digestive tract cancer by means of VOC analysis in exhaled breath. The aim was to examine the diagnostic performance of VOC analysis and also to identify potential pitfalls in order to improve future research in this field.

## Methods

### Search strategy

An electronic search of PubMed and the Cochrane Library was performed in May 2019. Neoplasm, cancer, tumour, electronic nose, volatile organic compounds, VOC, exhaled breath, predictive value of tests, sensitivity, and specificity were used as search terms, and were combined using AND–OR combinations.

Studies of cancer diagnosis that met the following criteria were included: at least two different groups of patients were included in the study, with regard to the presence of cancer; the index test was analysis of endogenous VOCs in exhaled breath; and the disease type was cancer of the digestive tract (oesophagus, stomach, liver, pancreas, and bowel). Studies were excluded if they were published before 2000, were not performed in adult humans, did not analyse malignant diseases, or analysed biofluids (such as breath condensate, urine, blood, and faeces).

The selection of potentially eligible articles was performed according to the PRISMA guidelines^28^. Discrepancies between the selections were solved in a consensus meeting between the reviewers. The following information was gathered independently and tabulated from the articles by type of cancer: author(s), year of publication, index test, reference test, method of data analysis, comparison groups, sensitivity, specificity, accuracy, and area under the curve (AUC). All VOCs identified in the studies were tabulated.

### Quality assessment

The methodological quality of the articles was assessed by means of the Quality Assessment of Diagnostic Studies 2 tool (QUADAS-2)^29^; a modified version was used^30^ (*Table S1*). The assessment was performed by two independent researchers and discrepancies were resolved by consensus.

**Table 1:** Quality assessment for each article

Reference	Risk of bias				Concerns regarding applicability		
	Patient selection	Index test	Reference standard	Flow and timing	Patient selection	Index test	Reference standard
**Gastro-oesophageal cancer**							
Abela *et al.*^40^	+	–	–	–	+	–	+
Amal *et al.*^32^	+	+	+	+	+	+	+
Amal *et al.*^31^	+	–	+	?	+	+	+
Chen *et al.*^33^	+	–	+	?	+	+	+
Daniel and Thangavel^34^	+	?	+	+	+	?	+
Duran-Acevedo *et al.*^35^	?	–	+	?	+	+	+
Kumar *et al.*^42^	–	–	+	+	+	+	+
Kumar *et al.*^41^	+	+	+	+	+	+	+
Markar *et al.*^43^	–	+	+	+	+	+	+
Schuermans *et al.*^36^	+	+	+	–	+	+	+
Shehada *et al.*^37^	?	+	?	?	+	+	?
Tong *et al.*^38^	?	?	?	?	+	–	?
Xu *et al.*^39^	–	+	+	?	+	+	+
Zou *et al.*^44^	?	–	?	?	+	+	?
**Colorectal cancer**							
Altomare *et al.*^49^	+	+	+	?	+	+	+
Altomare *et al.*^48^	–	–	+	–	–	+	?
Amal *et al.*^50^	+	+	+	+	+	+	+
Peng *et al.*^51^	–	–	?	–	+	–	+
van de goor *et al.*^52^	+	+	+	?	–	+	+
Wang *et al.*^53^	–	–	+	+	+	+	+
**Liver cancer**							
Qin *et al.*^45^	?	–	+	–	?	+	+
**Pancreatic cancer**							
Markar *et al.*^46^	+	+	+	+	+	+	+
Princivalle *et al.*^47^	?	–	?	–	+	+	+

+, Low risk; –, high risk; ?, unclear risk.

## Results

**Fig. 1:**
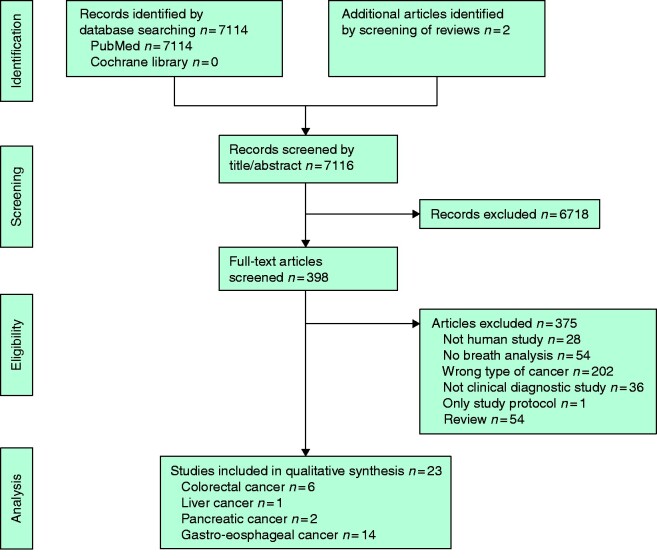
PRISMA diagram showing selection of articles for review

A total of 7114 studies were identified by the search in PubMed. After applying the eligibility criteria 21 articles were identified. Two articles were retrieved by manual search, and finally 23 articles^31–53^ were included in the review (*Fig. 1*).

### Quality assessment of the studies

**Fig. 2:**
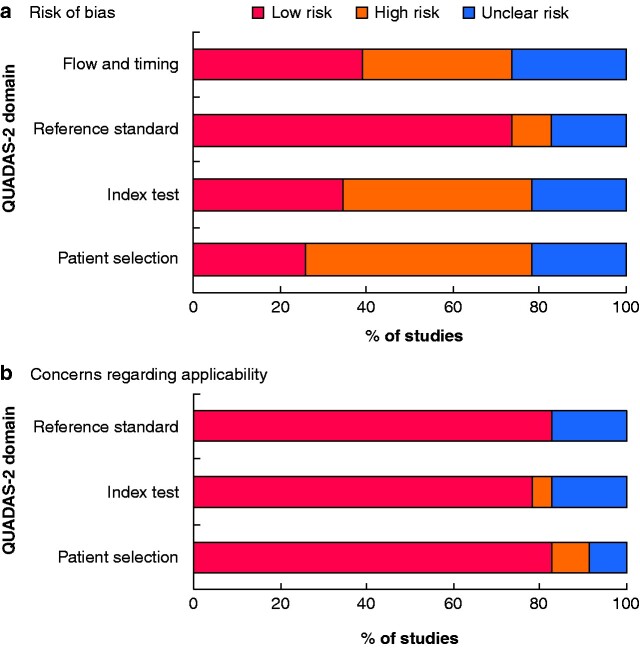
Summary of risk of bias and concerns regarding applicability for included studies **a** Risk of bias and **b** concerns regarding applicability.

An overview of the results of quality assessment is provided in *Table 1* and *Fig. 2*. The risk of bias was highest for patient selection. The most common reasons for unclear or high risk of bias were unclear specification, or issues regarding the eligibility criteria. For the index test criterion, the most common reason for a high-risk assessment was not having performed a blinded validation of the diagnostic model.

For flow and timing, the most common reason for high risk of bias was not having attempted to limit exogenous and endogenous influences on VOC composition. Regarding the applicability of the studies to the study question, the overall applicability concern was scored tolerantly and assessed as relatively low.

### Study characteristics

Fourteen articles describing studies in gastro-oesophageal cancer were included. The cancer population size ranged from 14 to 162 patients. Most studies (9) looked only at patients with gastric cancer^31–39^, whereas four studies^40–43^ included mixed oesophagogastric cancer. One study^44^ included only patients with oesophageal cancer. In most studies, the diagnosis was proven histologically; however, in four studies^37^^,^^38^^,^^40^^,^^44^, oesophagogastroduodenoscopy to rule out malignancy was not performed in controls.

Only one study^45^ included patients with liver cancer (30 patients). The patients had histologically proven stage I–V cancer and were compared with a group of healthy volunteers, and with a group of patients with hepatitis B-induced liver cirrhosis. The two control groups did not receive the same reference test as the cancer group. Patients with hepatitis B and cirrhosis were untreated and the disease confirmed histologically or cytologically. The healthy volunteers, however, who were the patient’s relatives and hospital staff with no history of cancer or other chronic disease, did not undergo any reference test.

Two studies^46^^,^^47^ included patients with histologically proven pancreatic cancer (25 and 65 patients respectively). The control groups consisted of perceived healthy controls in one study^47^, and patients suspected to have pancreatic disease who were scheduled for pancreatic imaging and found to be negative for malignancy in the other^46^.

Six studies^48–53^ analysed breath samples from patients with colorectal cancer. The study population ranged from 20 to 65 patients with cancer. Patients with stage I–IV disease were included in all but one study^48–52^; the other study^53^ included only patients with stage I–III tumours. The control group consisted of healthy controls in four studies^49–51^^,^^53^. One study^52^ compared VOCs from patients with colorectal cancer with those from patients with head and neck cancer (squamous cell carcinoma) or breast cancer. The remaining study^48^ was a follow-up analysis in which patients with colorectal cancer were compared with those with colorectal cancer from the original study^49^, who meanwhile had been treated and declared tumour-free. In addition, the follow-up patients were compared with healthy controls. All studies used histologically proven colorectal cancer as reference.

In general, many factors were heterogeneous across the studies. The eligibility criteria were sometimes not described clearly. Some studies included benign disease, whereas this was an exclusion criterion in other studies. There was also no consensus regarding how to deal with co-morbidities, and the timing of the index test compared with the reference test was not always at the same stage of the diagnostic process.

### Patient preparation and sample collection

Measures to reduce influences of ambient air were taken in 21 of 23 studies (*Table S2*). Performing a lung washout was done in 10 of 23 studies, and sampling ambient air as a reference value was performed in 9 of 23. Nineteen of 23 studies described having taken measures to limit the influence of food and/or beverages. The timing of fasting before breath collection ranged from 2 h to more than 24 h. Withholding from alcohol consumption and/or smoking before measurement was mentioned explicitly in 10 of 23 studies, and was at least recorded in 17 of 23 studies. Other preparatory measures described were withholding from physical exercise, being in an emotional balance, gurgling with water before breath collection, and restraining from the use of toothpaste.

**Table 2:** Overview of included articles by cancer type

Reference	Analytical platform and data analysis	Reference test	Cancer type and stage, and group size	Sensitivity (%)	Specificity (%)	Accuracy (%)	AUC
**Gastro-oesophageal cancer (*n* = 14)**							
Abela *et al.*^40^	Ultrasensitive TDLS; Mann–Whitney *U* test and Kruskal–Wallis test	TG: histology CG: selected from University of Glasgow database for ethane levels in healthy adults, without history of GI tumours	OC/GaC stage II–IV (*n* = 20) and HC (*n* = 10)	n.a.	n.a.	n.a.	n.a.
Amal *et al.*^32^	GC-MS; *t* test Cross-reactive nanoarrays (GNPs and SWCNTs covered with different ligands); DFA	TG: morphologically confirmed adenocarcinomas CG: upper endoscopy	Training phase 1) GaC stage I–IV (*n* = 69) and OLGIM 0–IV (*n = *230) 2) GaC stage I–IV (*n = *69) and OLGIM 0 (*n = *109) 3) GaC stage I–IV (*n = *69) and OLGIM 0–II (*n = *204) 4) GaC stage I–IV (*n = *69) and OLGIM III–IV (*n = *24) 5) GaC stage I–IV (*n = *69) and OLGIM I–IV (*n = *120) 6) GaC stage I–IV (*n = *69) and PUD (*n = *38) Validation phase 1) GaC stage I–IV (*n = *30) and OLGIM 0–IV (*n = *95) 2) GaC stage I–IV (*n = *30) and OLGIM 0 (*n = *46) 3) GaC stage I–IV (*n = *30) and OLGIM 0–II (*n = *87) 4) GaC stage I–IV (*n = *30) and OLGIM III–IV (*n = *10) 5) GaC stage I–IV (*n = *30) and OLGIM I–IV (*n = *50) 6) GaC stage I–IV (*n = *30) and PUD (*n = *30)	Training phase 1) 84 2) 93 3) 94 4) 96 5) 94 6) 93 Validation phase 1) 73 2) 90 3) 97 4) 93 5) 93 6) 87	Training phase 1) 86 2) 84 3) 91 4) 83 5) 95 6) 89 Validation phase 1) 98 2) 80 3) 84 4) 80 5) 80 6) 87	Training phase 1) 85 2) 88 3) 92 4) 92 5) 95 6) 92 Validation phase 1) 92 2) 84 3) 87 4) 90 5) 85 6) 87	n.a.
Amal *et al.*^31^	TD-GC-MS; Wilcoxon/Kruskal–Wallis test	TG: upper endoscopy + histology CG: upper endoscopy	China 1) GaC stage I–IV (*n = *37) and control (combined) (*n = *61) Latvia 2) GaC stage I–IV (*n = *37) and control (combined) (*n = *61)	n.a.	n.a.	n.a.	n.a.
Chen *et al.*^33^	SPME-GC-MS; *t* test SERS sensor; ANOVA (Diagnostic tool developed based on simulated breath samples and validated on real patients)	TG: gastroscopy + histology CG: endoscopy + histology	1) EGC (*n = *55) and control (*n = *56) + AGC (*n = *89) 2) AGC (*n = *89) and control (*n = *56) + EGC (*n = *55)	1) 87.3 2) 89.9	1) 94.1 2) 92.0	n.a.	n.a.
Daniel and Thangavel^34^	3 metal oxide semiconductor gas sensor arrays (TGS813, TGS822, TGS2620) (Figaro); ANN—CFBP and FFBP (Diagnostic model trained with 90 per cent of data and 10 per cent used for validation)	TG: gastroscopy and biopsy when abnormalities discovered CG: gastroscopy	GaC (*n = *49) *versus* controls (mix) (*n = *112)	94.4	89.9	93	n.a.
Duran-Acevedo *et al.*^35^	SPME-GC-MS (GC/Q-TOF); PCA And/or chemical gas sensor with AGD; PCA	TG: gastroscopy + histology CG: gastroscopy (+ histology)	GC-MS analysis 1) GaC (*n = *14) and controls (mix) (*n = *16) Chemical sensor analysis 2) CG (*n = *11) and controls (mix) (*n = *16)	GC-MS analysis 1) 93 Chemical sensor analysis 2) 100	GC-MS analysis 1) 87 Chemical sensor analysis 2) 93	GC-MS analysis 1) 90 Chemical sensor analysis 2) 97	n.a.
Kumar *et al.*^42^	SIFT-MS + MIM; Mann–Whitney *U* test and LLR	TG: histology CG: OGD	Diagnostic model based on 4 VOCs OC/GaC (*n = *18) and control (benign) (*n = *18)	n.a.	n.a.	n.a.	0.91
Kumar *et al.*^41^	SIFT-MS; Mann Whitney *U* test (Diagnostic model based on binary LLR; 2/3 for model development, 1/3 for validation with accuracy based on mean of 10× monte Carlo simulations)	TG: OGD + histology CG: OGD	VOC data analysis 1) GaC stage I–III (*n = *33) and HC (*n = *62) 2) GaC stage I–III (*n = *33) and control (mix) (*n = *129) 3) OC stage I–III (*n = *48) and HC (*n* = 62) 4) OC stage I–III (*n = *48) and control (mix) (*n = *129) Diagnostic prediction model: training phase 5) GaC/OC stage I–III (*n = *53) and control (mix) (*n = *85) Diagnostic prediction model: validation phase 6) GaC/OC stage I–III (*n = *28) and control (mix) (*n = *44)	VOC data analysis 1) 100 2) 87.9 3) 98 4) 87.5 Diagnostic prediction model: training phase 5) 89.3 Diagnostic prediction model: validation phase 6) 86.7	VOC data analysis 1) 92.2 2) 88.5 3) 91.7 4) 82.9 Diagnostic prediction model: training phase 5) 83.7 Diagnostic prediction model: validation phase 6) 81.7	n.a.	VOC data analysis 1) 0.98 2) 0.92 3) 0.97 4) 0.90 Diagnostic prediction model; training phase 5) 0.92 Diagnostic prediction model: validation phase 6) 0.87
Markar *et al.*^43^	SIFT-MS (+ cross validation with GC-MS) (5-VOC-predictive model based on multivariable LLR)	TG: histologically proven OG cancer (non-metastatic) CG: gastroscopy	OC/GaC stage I–IV (*n = *162) and controls (benign mix) (*n = *163)	80	81	n.a.	0.85
Schuermans *et al.*^36^	AEONOSE; ANN	TG: after confirmed tumour diagnosis CG: family members screened by endoscopy and negative for gastric malignancies	GaC (*n = *16) and HC (*n = *28)	81	71	75	0.83
Shehada *et al.*^37^	TPS-SiNW FET (individually modified); DFA (Diagnostic model based on DFA with 75 per cent of samples used as training set and 25 per cent for blinded validation)	n.a.	Training phase 1) GaC stage I–IV (*n = *22) and control (mix) (*n = *58) Validation phase 2) GaC stage I–IV (*n = *8) and control (mix) (*n = *19)	Training phase 1) 87 Validation phase 2) 71	Training phase 1) 81 Validation phase 2) 89	Training phase 1) 83 Validation phase 2) 85	n.a.
Tong *et al.*^38^	SPME-GC-MS; PCA, PLSDA (with VIP) and two-sided Welch 2-sample *t* test	n.a.	1) GC (*n = *24) and HC (*n = *32) 2) GC (*n = *24) and PUD (*n = *24) 3) CG (*n = *24) and gastritis (*n = *48)	n.a.	n.a.	n.a.	n.a.
Xu *et al.*^39^	GC-MS; Wilcoxon Kruskal–Wallis test Nanomaterial-based sensor array; DFA (training on 100 per cent of samples, validation on blinded 25 per cent of samples)	TG: endoscopy and histology CG: endoscopy (+biopsy)	1) GaC stage I–IV (*n = *37) and control (benign mix) (*n = *93) 2) GaC stage I–IV (*n = *37) and GU (*n = *32) and control (benign) (*n = *61) 3) GaC stage I–IV (*n = *6) and control (benign mix) (*n = *26)	Training phase 1) 89 Validation phase 3) 83	Training phase 1) 90 Validation phase 3) 96	Training phase 1) 90 2) 77 Validation phase 3) 94	n.a.
Zou *et al.*^44^	Home-made PTR-MS (Ion Sniffer 2020Q); Mann–Whitney *U* test and SDA	TG: diagnosed with OC, not further specified CG: not specified	SDA analysis based on 20 VOCS 1) OC stage I–IV (*n = *29) and HC (*n = *57) 2) OC stage I (*n = *1) and HC (*n = *57) 3) OC stage II (*n = *7) and OC (*n = *57) 4) OC stage III (*n = *7) and HC (*n = *57) 5) OC stage IV (*n = *14) and HC (*n = *57) ROC analysis using 7 VOCs 6) OC stage I–IV (*n = *29) and HC (*n = *57)	SDA analysis based on 20 VOCS 1) 86.2	SDA analysis based on 20 VOCS 1) 89.5	SDA analysis based on 20 VOCS 2) 100 3) 71 4) 86 5) 93	ROC analysis 6) 0.943%
**Liver cancer (*n = *1)**							
Qin *et al.*^45^	SPME-GC-MS; Mann–Whitney *U* test (Diagnostic model based on Fisher linear discriminant functions, cross-validation and leave-1-out procedure)	TG: cytology or histology CG (cirrhosis): clinically diagnosed with hepatocirrhosis induced by hepB virus CG: relatives and hospital staff	Per VOC analysis in different groups HCC stage I–IV + hepB (*n = *30) and HC (*n = *36) 1) 3-Hydroxy-2-butanone 2) Styrene 3) Decane HCC stage I–IV +HepB (*n = *30) and cirrhosis +HepB (*n = *27) 4) 3-Hydroxy-2-butanone 5) Styrene 6) Decane Diagnostic model 7) HCC stage I–IV + HepB (*n = *30) and HC (*n = *36)	Per VOC analysis HCC and HC 1) 83.3 2) 66.7 3) 86.7 HCC and cirrhosis 4) 70.0 5) 66.7 6) 76.7 Diagnostic model 7) 86.7	Per VOC analysis HCC and HC 1) 91.7 2) 94.4 3) 58,3 HCC and cirrhosis 4) 70.4 5) 70.4 6) 48.1 Diagnostic model 7) 91.7	n.a.	Per VOC analysis HCC and HC 1) 0.926 2) 0.812 3) 0.798 HCC and cirrhosis 4) 0.745 5) 0.686 6) 0.637
**Pancreatic cancer (*n = *2)**							
Markar *et al.*^46^	TD-GC-MS; Mann– Whitney *U* test and LLR	TG: CT abdomen/endoscopic ultrasonography + histologically by FNA CG: recruited with a pancreatic condition or other patients scheduled for pancreatic ultrasonography or abdominal CT, included when negative on imaging	Training phase 1) PC (mix) (*n = *25) and no cancer mix (*n = *43) 2) AC (*n = *17) and no cancer mix (*n = *43) 3) AC, local (*n = *7) and no cancer (*n = *43) Validation phase 4) PC (mix) (*n = *32) and no cancer mix (*n = *32) 5) AC (*n = *28) and no cancer mix (*n = *32) 6) AC, local (*n = *14) and no cancer (*n = *32)	Training phase 1) 80 2) 94 3) 100 Validation phase 4) 81 5) 70 6) 79	Training phase 1) 95 2) 91 3) 100 Validation phase 4) 51 5) 74 6) 81	n.a.	Training phase 1) 0.901 (0.819-0.982) 2) 0.99 (0.973-1.00) 3) 0.10 Validation phase 4) 0.736 (0.614-0.858) 5) 0.744 (0.615-0.873) 6) 0.855 (0.732-0.914)
Princivalle *et al.*^47^	IMR-MS (AirSense analyser; V&F); LASSO and LLR (Diagnostic model based on age + 10 VOCs)	TG: cytohistology CG: perceived healthy controls	PDA (*n = *65) and HC (*n = *102)	Diagnostic model 100	Diagnostic model 84.3	n.a.	Diagnostic model 0.987
**Colorectal cancer (*n = *6)**							
Altomare *et al.*^49^	GC-MS; PNN	TG: histology CG: colonoscopy Validation phase: not specified	Trial phase 1) CRC stage I–IV (*n = *37) and HC (*n = *41) Validation phase 2) CRC stage I–IV (*n = *15) and HC (*n = *10)	Trial phase 86 (71, 95)^*^ Validation phase 2) n.a.	Trial phase 1) 83 (68, 93)^*^ Validation phase 2) n.a.	Trial phase 1) 85 Validation phase 2) 76	Trial phase 1) 0.85.2
Altomare *et al.*^48^	TD-GC-MS; PNN and Mann– Whitney *U* test	TG: histology CG: colonoscopy	31-VOC model 1) CRC stage I–IV (*n = *48) and CRC-FU (*n = *32) 2) CRC-FU (*n = *32) and HC (*n = *55) 11-VOC model (overlapping VOCs with previous study) 3) CRC stage I–IV (*n = *48) and CRC-FU (*n = *32) 4) CRC-FU (*n = *32) and HC (*n = *55)	31-VOC model 1) 100 (92.6, 100)^*^ 2) 100 11-VOC model 3) 100 4) 100	31-VOC model 1) 95.8 (83.8, 99.9)^*^ 2) 96.4 11-VOC model 3) 97.9 4) 90.9	31-VOC model 1) 97.5 2) 97.7 11-VOC model 3) 98.8 4) 94.3	31-VOC model 1) 0.993 2) 0.992 11-VOC model 3) 0.10 4) 0.959
Amal *et al.*^50^	GC-MS; Student’s t test Cross reactive nanoarrays; DFA	TG: histology CG: negative medical history and colonoscopy	Training phase 1) CRC stage I–IV (*n = *45) and HC (*n = *86) 2) CRC stage I–IV (*n* = 45) and adenoma (*n = *22) 3) HC (*n = *86) and adenoma (*n = *22) 4) NAA (*n = *10) and AA (*n = *12) Validation phase 5) CRC stage I–IV (*n = *20) and HC (*n = *36) 6) CRC stage I–IV (*n* = 16) and adenoma (*n = *16) 7) HC (*n = *16) and adenoma (*n = *16) 8) NAA (*n = *8) and AA (*n = *8)	Training phase 1) 93 (82, 98)^*^ 2) 95 3) 94 4) 100 Validation phase 5) 94 (62, 97)^*^ 6) 88 7) 94 8) 88	Training phase 1) 88 (89, 99)^*^ 2) 90 3) 94 4) 89 Validation phase 5) 91 (81, 99)^*^ 6) 91 7) 94 8) 94	Training phase 1) 90 2) 92 3) 94 4) 95 Validation phase 5) 91 6) 91 7) 94 8) 94	n.a.
Peng *et al.*^51^	GNP sensor array; PCA SPME-GC-MS, ADMIS	TG: imaging and histology CG: n.a. Exclusions: n.a.	CRC stage I–IV (*n = *26) and HC (*n = *22)	n.a.	n.a.	n.a.	n.a.
van de Goor *et al.*^52^	AEONOSE; ANN	TG: histology CG: histology	1) CRC stage I–IV (*n = *28) and HNSCC stage 0–IV (*n = *100) 2) CRC stage I–IV (*n = *28) and BC stage 0–IV (*n = *40)	1) 79 2) 88	1) 81 2) 79	1) 83 2) 84	1) 0.83 (0.74-0.92) 2) 0.90 (0.81-0.98)
Wang *et al.*^53^	SPME-GC-MS; PCA and PLSDA	TG: histology CG: negative medical history and colonoscopy	CRC stage I–III (*n = *20) and HC (*n = *20)	n.a.	n.a.	n.a.	n.a.

* Values in parentheses are 95 per cent confidence interval. AUC, area under the curve; TDLS, tuneable diode laser spectrometer; TG, test group; CG, control group; GI, gastrointestinal; HC, healthy controls; OC, oesophageal cancer; GaC, gastric cancer; n.a., not available; GC-MS, gas chromatography–mass spectrometry; GNP, gold nanoparticles; SWCNT, single-wall carbon nanotube; DFA, discriminant function analysis; OLGIM, operative link on gastric intestinal metaplasia; PUD, peptic ulcer disease; TD-GC-MS, thermal desorption GC-MS; SPME-GC-MS, solid-phase micro extraction GC-MS; SERS, surface enhanced Raman scattering; EGC, early gastric cancer; AGC, advanced gastric cancer; ANN, artificial neural network; CFBP, cascade forward back propagation; FFBP, feed forward back propagation; GC/Q-TOF, quadrupole time-of-flight gas chromatography mass spectrometry; PCA, principal component analysis; AGD, advanced gas deposition system; SIFT-MS, selected ion flow tube mass spectrometry; MIM, multiple ion monitoring; LLR, logistic regression; VOC, volatile organic compound; OGD, oesophagogastric duodenoscopy; TPS-SiNW FET, trichloro(phenethyl)silane field effect transistor; PLSDA, partial least squared discriminant analysis; VIP, variable importance in the projection; GU, gastric ulcer; PTR-MS, proton transfer reaction mass spectrometry; SDA, stepwise discriminant analysis; ROC, receiver operating characteristic; hepB, hepatitis B; HCC, hepatocellular carcinoma; FNA, fine-needle aspiration; PC, pancreatic cancer; AC, adenocarcinoma; IMR-MS, ion molecule reaction mass spectrometry; LASSO, least absolute shrinkage and selection operator; PDA, pancreatic ductal adenoma; PNN, probabilistic neural network; CRC, colorectal cancer; CRC-FU, follow-up 1 year after CRC surgery (+ chemotherapy); AA, advanced adenoma; NAA, non-advanced adenoma; ADMIS, automated mass spectral deconvolution and identification system; HNSCC, head and neck squamous cell carcinoma; BC, breast cancer.

The timing of breath collection in the diagnostic process differed between the studies. Sample collection was performed using the following systems: Mylar^®^ bags, Tedlar^®^bags, syringes, inert steel bags or chambers, nalophan sampling bags, BioVOC^tm^ breath sampler, directly into PTR-MS instrument, and directly into e-nose. Research groups then stored and analysed the samples themselves or transported them to a laboratory that had access to the required analytical platform.

### Analytical platforms and data analysis

A variety of methods were used to analyse VOCs from exhaled breath (*Table 2*). GC-MS and sensor array systems were most often used to analyse exhaled breath (8 studies)^32^^,^^33^^,^^35^^,^^39^^,^^43^^,^^46^^,^^50^^,^^51^, followed GC-MS alone (6 studies) ^31^^,^^38^^,^^45^^,^^48^^,^^49^^,^^53^. Other systems used were: SIFT-MS (2 studies)^41^^,^^42^, home-made PTR-MS (1)^44^, ultrasensitive tuneable diode laser spectrometer (1 study)^40^, trichloro(phenethyl)silane field effect transistor (1 study)^37^, and IMR-MS (1 study)^47^. Only three studies^34^^,^^36^^,^^52^ used sensor systems for analysis: AEONOSE (eNose company) (2 studies) and breath analyser (Figaro, USA) (1 study).

Data analysis was performed by a variety of techniques, involving the following methods: principal component analysis (PCA), probabilistic neural networks, partial least squared discriminant analysis (PLSDA), discriminant function analysis, artificial neural networks, Fisher least discriminant function analysis, least shrinkage and selection operator logistic regression (LLR), Mann–Whitney *U* test with LLR, predictive probability models, Mann–Whitney *U* test with binary logistic regression model, PCA with PLSDA with variable importance in the projection model, *t* test with ANOVA, and PCA with stepwise discriminant analysis. A detailed explanation of these methods is beyond the scope of this review.

### Diagnostic test performance and validation

A summary of the diagnostic performance of the individual studies is provided in *Table 2*. The results were divided into four groups based on the cancer type studied. Data on sensitivity, specificity, accuracy, and AUC were retrieved from the articles. Where a study compared the index group with multiple reference groups, the results of these comparisons are also included. Five authors did not report diagnostic performance. The sensitivity ranged from 66.7 to 100 per cent, whereas specificity ranged from 48.1 to 97.9 per cent. In most studies, the sensitivity and specificity were lower in the validation phase than the training phase. Owing to heterogeneity of the studies, no meta-analysis could be performed. Internal, external or cross-validation was performed in one third of the studies (*Table 2*).

Both the largest (484 patients)^32^ and the smallest (30)^40^ studies, including patients and controls, analysed VOCs from patients with gastro-oesophageal cancer.

### Reported volatile organic compounds

In total, 106 different VOCs were identified. For most VOCs, there was a statistically significant difference in presence between the groups. Some of the identified VOCs were only significant within a subgroup. Of the VOCs recorded, 32 were identified by more than one study (*Table S3*). These VOCs were either found to be cancer type-specific in multiple studies (19 VOCs), or were found in different cancer types (13 VOCs) and were therefore more general cancer VOCs. The VOCs that were identified in the most studies (4 studies each) were decanal, nonanal, and acetone.

Seventeen studies reported on VOCs that were present at significantly different levels in the exhaled breath of patients with cancer and control groups. All 106 VOCs identified in the studies are summarized in *Table S4* (VOCs identified in multiple studies are highlighted in different colours). In total, 32 of the 106 VOCs were present differently in multiple studies. Ranked from high to low based on number of studies they were mentioned in, these were: decanal (4), nonanal (4), acetone (4), 1,3-dimethylbenzene (3), 2-methylpentane (3), 3-methylpentane (3), 2-propenenitrile (3), furfural (3), 4-methyloctane (3), isoprene (3), 1,2-pentadiene (2), 1,4-dimethylbenzene (2), 1,2,3-trimethylbenzene (2), undecande (2), dodecane (2), 4-methyl-2-pentanone (2), hexane (2), cyclohexane (2), methylcyclopentane (2), methylcyclohexane (2), ammonia (2), pentane (2), tetradecane (2), butanal (2), butyric acid (2), hexaoic acid (2), pentanoic acid (2), 2-butoxy-ethanol (2), 6-methyl-5-hepten-2-one (2), methanol (2), ethyl phenol (2), hexadecane (2).

Thirteen compounds identified in multiple studies were described for different cancer types. Acetone was found to be significantly different in the oesophageal cancer/gastric cancer, pancreatic cancer, and colorectal cancer groups. 2-Methylpentane, 3-methypentane, 4-methyloctane, dodecane, decanal, and nonanal were found in the oesophageal cancer/gastric cancer and colorectal cancer groups. Pentane,undecane,tetradecane, hexane, ammonia and 1,2,3-trimethylbenzene were found in the pancreatic cancer and colorectal cancer groups. The remaining 19 VOCs were found only in studies of the same cancer.

## Discussion

The diagnostic performance of breath analysis for diagnosing cancer has shown promising results, with good sensitivity and specificity. The potential use of breath analysis as a non-invasive test that can be applied clinically may differ for each specific type of digestive tract malignancy as it depends on the cancer prevalence and existing diagnostic alternatives. Breath analysis could be considered as an additional screening tool to supplement faecal blood testing in colorectal cancer, or a screening tool for gastric cancer in countries with a high incidence, such as Asian countries, including Japan. Another option could be monitoring of patients with Barrett’s oesophagus to detect a potential conversion to malignancy. Breath analysis might be of special interest for pancreatic cancer, as its incidence is rising and the prognosis is poor, partly because it is often missed in the early stages^14^. A non-invasive test with the ability to distinguish between benign and malignant masses would be welcome. Despite the amount of research already done, there is currently no breath test being used for the detection of gastrointestinal tract malignancies, and the majority of clinical investigations are proof-of-concept studies. Most of these studies have been performed in small populations using different analytical techniques with poor standardization. VOCs are a product of metabolic processes and so their presence in exhaled breath greatly depends on the metabolic state of the patient. Alterations in breath profiles could not only be induced by cancer but also by other potential endogenous and exogenous influences, such as fasting status, microbiome, smoking, medication, co-morbidities, and exposure to varying ambient air pollutants; all these issues should be taken into consideration when designing a diagnostic study on breath analysis^21^.

Several initiatives are under way to develop protocols for standardization of sampling and analytical measurements in the International Association of Breath Research^54–56^ and the European Respiratory Society^57^. In a recent review^30^, a proposed framework for conducting and reporting future studies investigating the role of VOCs in cancer diagnosis was formulated. Applying standardization would contribute to improved quality of individual studies and enhance comparison between studies, leading to faster implementation of this promising diagnostic tool in clinical practice.

Although there is an abundance of possibilities for performing VOC analysis, a disadvantage in most of the currently available studies is possible overestimation of the predictive value and lack of external validation. Prediction models generally perform better on data on which the model was developed than on new data. Owing to relatively small sample sizes in most of the studies, there is a lack of external validation leading to a possible reduction in reproducibility^58^. According to the TRIPOD statement^59^, it is highly recommended for studies of prediction models to at least perform internal validation of the findings. Truly reliable results will only be generated by also validating the results externally.

There are many different analytical methods being used in studies of VOCs, and a distinction can be made between the so called real-time and offline analysis techniques^22^. The majority of the included studies used an offline combination of GC-MS systems with a sensor array system. An advantage of this approach is that specific discriminative VOCs can be identified and used to develop sensor systems applicable to clinical settings. However, certain conditions must be fulfilled for development of a breath test for use in the clinic. For clinical use, it is most important that the device is easy to carry, gives quick results, is non-invasive, should not be susceptible to environmental influences, and has both a high sensitivity and specificity.

VOCs that appeared in multiple studies might have the most discriminative value for discriminating cancer from non-cancer conditions. Some VOCs, such as acetone, 2-methylpentane, 3-methylpetane, decanal, nonanal, pentane, and tetradecane, were identified in studies of different cancer types. This suggests that VOCs can be cancer type-specific, but also general markers for cancer. The vast majority of the VOCs, however, were only identified in single studies. Of the single VOCs that were identified in multiple studies, including decanal, nonanal and acetone, not all can be attributed directly to certain (patho)physiological processes. However, it is known that cancers often show metabolic abnormalities, such as dysregulation of glucose, fatty acid, and amino acid metabolism^60^. One should keep in mind that not only cancers but also other metabolic abnormalities might cause alterations in breath profiles. For example, an increase in acetone can be a result of diabetic ketoacidosis. However, acetone is a ketone strongly related to fatty acid oxidation. Fatty acids consist of a carboxyl group and a hydrocarbon chain that can be saturated or unsaturated, and are required for synthesis of membranes and signalling molecules in cellular proliferation, as seen in cancers^60–62^.

Headspace analysis of healthy intestinal epithelial cells and colonic cancer cells has already shown differences in release of VOCs. This indicates that metabolic abnormalities of cancer cells might contribute to the differences in exhaled breath profiles^63^. As the pathophysiological mechanisms that lead to the altered VOC production in patients with cancer have not yet been elaborated sufficiently, it remains difficult to determine the origin of the distinctive VOCs.

More recent studies using sensor systems, such as the Aenose, have shown promising results of exhaled breath analysis for diagnosing malignancies. However, these studies were unable to identify individual compounds as they used sensor measurements that were analysed using pattern recognition techniques^64^. Additionally, they can be criticized for showing poor linear reproducibility of the results and they also seem to be particularly sensitive to exogenous influences, such as humidity^17^.

As for use in clinical practice, it would be of interest to determine whether a breath test could be applied not only to distinguish between healthy patients and those with cancer, but also between similar diseases such as cancer and benign conditions of the same organ^22^. Therefore, one should consider also including patients with benign diseases in breath analysis studies. During the review process, an additional study^65^ was published that met the search criteria for the present analysis. Breath analysis was performed using the Aenose for diagnosing colorectal cancer. The final model for distinguishing colorectal cancer from healthy controls showed a sensitivity of 95 per cent and specificity of 64 per cent, with an AUC of 0.84. Benign conditions such as advanced adenoma, non-advanced adenomas or hyperplastic polyps were also taken into account. Although the Aenose was able to distinguish patients with colorectal cancer from healthy controls, it was not able to differentiate colorectal cancer from advanced adenomas, or advanced adenomas from non-advanced adenomas, suggesting that the VOC profiles are too similar^65^. A different study^66^ using the Aenose for a known precursor of oesophageal carcinoma, Barrett’s oesophagus, had shown promising results, with a sensitivity of 91 per cent and specificity of 74 per cent for differentiating patients with Barrett’s oesophagus from healthy controls. These findings demonstrate that exhaled breath analysis may be of use in the early detection of precancerous conditions, enabling better surveillance or earlier treatment. However, as discussed above, a number of steps still need to be taken to develop clinically applicable breath tests.

Currently, multiple systems are used for VOC detection, which have similar diagnostic performance. However, comparison and pooling of the studies proved to be difficult in the present analysis owing to wide heterogeneity between the studies. A consensus on how studies that analyse VOCs in exhaled breath should be performed will greatly advance progress in this field.

The appearance of some of VOCs in multiple studies of the same cancer type, but also different cancer types, suggests that there could be tumour-specific and also general cancer-associated VOCs. Further studies are needed to determine whether such VOCs could be used to improve cancer diagnostics.

## Funding

This study was supported by the Dutch Digestive Foundation (MLDS career development grant CDG16-12 to T.L.)

Conflict of interest.

The authors declare no conflict of interest.

## Supplementary material

Supplementary material is available at *BJS Open* online.

## Supplementary Material

zrab013_Supplementary_Data
