# Supplementary material for: Breath analysis for the detection of digestive tract malignancies: systematic review
Source: BJS Open. 2021 Apr 15;5(2):zrab013. doi: 10.1093/bjsopen/zrab013 (PMC8047095; doi:10.1093/bjsopen/zrab013)
Supplement: zrab013_Supplementary_Data [file zrab013_supplementary_data.zip › Supplement Tables 1-4 R3 version.docx]

***Appendix***

**Supplement Table 1: QUADAS-2 tool with extra modifications**

|  |  | **QUADAS-2** | **QUADAS-2 (modified)** | **Type of modification** |
| --- | --- | --- | --- | --- |
| **RISK of BIAS** | **Patient selection** | Was a consecutive or random sample of patients enrolled? | Were sampled patients representative of the intended population?  Info: Patients that came to the hospital for either diagnosis of complaints, or took part in a screening program | **(Am)** |
|  |  | Was a case-control design avoided? | Did the study include both positive (benign conditions) as well as healthy controls? | **(Am)** |
|  |  | Did the study avoid inappropriate exclusions? | Did the study avoid inappropriate exclusions?  Info: inappropriate was defined as: exclusions that the breath test is supposed to be able to differentiate the malignancy from, e.g. benign diseases of the organ, or premalignancies eg gastritis, IBS, barret esophagus, adenomas | **(Un)** |
|  | **Index test** | Were the index test results interpreted without knowledge of the results of the reference standard? | Was the index test and interpretation of data performed in standardised and reproducible fashion? | **(Am)** |
|  |  | If a threshold was used, was it pre-specified? | Validation of results performed (internal or external)?  Info: Did they validate their results on an blinded sample? | **(Am)** |
|  | **Reference standard** | Is the reference standard likely to correctly classify the target condition? | Is the reference standard likely to correctly classify the target condition? | **(Un)** |
|  |  | Were the reference standard results interpreted without knowledge of the results of the index test? | - | **(Om)*** |
|  |  | - | Is the reference standard likely to correctly classify the control group? | **(Extra modification)** |
|  | **Flow and timing** | Was there an appropriate interval between index test and reference standard? | Was there an appropriate interval between index test and reference standard?  Info: Ideally, prior to reference test. If not there should at least be enough time between the reference test and the index test, so that it does not influence the breath sample. | **(Un)** |
|  |  | - | Were index test and reference standard performed prior to therapeutic intervention? | **(Ad)** |
|  |  | Did all patients receive the same reference standard? | Did all patients receive the same reference standard? | **(Un)** |
|  |  | - | Were measures taken to limit exogenous influences such as food intake and smoking etc? | **(Extra modification)** |
|  |  | Were all patients included in the analysis? | Were all patients included in the analysis? | **(Un)** |
| **APPLICABILITY** | **Patient selection** | Are there concerns that the included patients and setting do not match the review question? | Are there concerns that the included patients and setting do not match the review question? | **(Un)** |
|  | **Index test** | Are there concerns that the index test, its conduct, or interpretation differs from the review question? | Are there concerns that the authors have not demonstrated suitable reproducibility and sensitivity of the chosen index test? | **(Am)** |
|  | **Reference standard** | Are there concerns that the target condition as defined by the reference standard does not match the question? | Are there concerns that the target condition as defined by the reference standard does not match the question? | **(Un)** |
|  | Am = amendment. Om = omitted. Ad = addition. Un = unchanged. Extra modification= modifications done by our research group.  *Criteria omitted as not applicable in the case of phase 1 biomarker discovery studies  **REVIEW QUESTION**  Test population: human subjects  Index test(s): VOC analysis within exhaled breath  Reference standard: the accepted standard for diagnosis of cancer and/or benign disease in that field  Target condition: cancer of the gastrointestinal tract (esophagus, stomach, liver, pancreas, small and large bowel)  Setting: hospital, medical centre  Intended use of the index test: diagnostic  Patient presentation: routine investigation for symptoms of malignancy  For this paper we made use of the modifications of the QUADAS-2 tool that had previously been done by Hanna et al. (2018). | | | |

**Supplement Table 2. Standardization methods for the sampling procedures in the retrieved studies**

| **Publication (Author, Year)** | **Room air** | **Food** | **Beverage** | **Alcohol** | **Smoking** | **Drugs** | **Excercise** | **Co-morbidities** | **Biopsy** | **Diagnosis** | **Treatment** | **Other** |
| --- | --- | --- | --- | --- | --- | --- | --- | --- | --- | --- | --- | --- |
| ***Gastro-Esophageal Cancer (n=14)*** | | | | | | | | | | | | |
| Abela et al.^40^, 2009 | >30min at clinic, room air sampled | NA | NA | NA | >30 min | NA | NA | NA | NA | After | Prior | NA |
| Amal et al.^32^, 2016 | Same room, 3 min lung washout, room air sampled | >12h/>2h | NA | >12h/>2h | >12H/>2h | No PPI or antibiotics/recorded | NA | H.pylori registered | After | After | NA | NA |
| Amal et al.^31^, 2013 | 3 min lung washout | >12 h | NA | Recorded | >3 h, smoking status recorded | PPI use recorded | NA | Helicobacter testes | Post | Post | Prior to surgery, chemo and/or radiotherapy; controls prior to upper endoscopy | NA |
| Chen et al.^33^,  2016 | Rested in same room 30 min prior to sampling | Overnight | NA | Overnight | Overnight | NA | >2d | No exclusions | After | After | NA | Emotional balance |
| Daniel et al.^34^, 2016 | Same clinical environment | >12 h | NA | >12 h | >12 h | No med affecting gastric secretions and/or antibiotics 1 minth prior to sampling | >1h, rest, >24 h no exercise | See exclusion criteria | After | After | NA | NA |
| Duran Acevedo et al.^35^, 2018 | NA | >10 h | >10 h | NA | NA | Recorded | NA | H.Pylori infection recorded | After | After endoscopy | Prior | Living zone recorded |
| Kumar et al.^42^, 2013 | Same room, room air sampled | >6 h | >6 h | NA | Recorded | NA | NA | Recorded | NA | EGD on day of sampling, not further spec | Prior | Patients seated during sampling |
| Kumar et al.^41^, 2015 | NA | >6h | NA | Recorded | Recorded | PPI use recorded | NA | Recorded | NA | Prior | Prior | NA |
| Markar et al.^43^, 2018 | Same environment for 20 min, 3 different hospitals? | >4h | Recorded | Recorded | Recorded | Recorded | NA | Recorded | Prior | Prior | Prior | NA |
| Schuermans et al.^36^, 2018 | 2 min of flushing by AENOSE | NA | NA | NA | NA | NA | NA | recorded | NA | After | Prior | Location of measurement recorded, complaints recorded |
| Shehada et al.^37^, 2015 | Same room, lung washout during 3 min | >2h | NA | >2h | >2h | NA | NA | H.pylori infection recorded | NA | NA | NA | NA |
| Tong et al.^38^, 2017 | Room air sampled in parallel | >24 h | NA | NA | Smoking status recorded | NA | NA | NA | NA | NA | NA | NA |
| Xu et al.^39^, 2013 | Sampling in clinical environment, 3 min lung washout | >12 h | NA | >12 | >12 h | NA | Rest 1 h before sampling, >24 h no exercise | NA | NA | NA | NA | NA |
| Zou et al.^44^, 2016 | Patient being at lab room 5 min prior to sampling, temperature of the room regulated | >10 h | >10 h | NA | NA | NA | NA | NA | NA | After | NA | Gurgle with water prior to measurement, >10 h no toothpaste |
| ***Liver Cancer (n=1)*** | | | | | | | | | | | | |
| Qin et al.^45^, 2010 | Ambient air collected for reference | Overnight | NA | Recorded | Recorded | NA | NA | NA | After | After | NA | NA |
| ***Pancreatic Cancer (n=2)*** | | | | | | | | | | | | |
| Markar et al.^46^, 2018 | Room air and laboratorium air sampled, charcoal filter to clean inspired air | >4h | NA | NA | >4h | NA | NA | Recorded | After | After | Before | NA |
| Princivalle et al.^47^, 2018 | BIO-VOC breath sampler with valve to prevent rebreathing,. Room air sampled. | TG: overnight  CG: >2h | TG: overnight  CG: >2h | Recorded | Recorded | NA | NA | Recorded (incl CA19-9) | After | After | 1-3 says before surgery | NA |
| ***Colorectal Cancer (n=6)*** | | | | | | | | | | | | |
| Altomare et al.^49^, 2013 | Same room, 10 min acclimatization, 5 min lung washout | >3 h | >3 h | NA | NA | recorded | NA | Recorded | After | After | Day of surgery | Before bowel preparation |
| Altomare et al.^48^, 2015 | Same room, 10 min acclimatization, 4 min lung washout | >3h | >3 h | NA | NA | NA | NA | NA | CRC group: After  FU group:  After  HC group: | After | CRC group: FU group: after | NA |
| Amal et al.^50^, 2016 | Room air sampled in parallel | Overnight fasting recomended, 15% postprandial sampling | Overnight fasting recomended, 15% postprandial sampling | Overnight fasting recomended, 15% postprandial sampling | >2h | NA | NA | Pt. with previously cured malignancies included | Before | Before | Before | Prior to coloscopy  Control group: 1 wk after colonoscopy |
| Peng et al.^51^, 2010 | Same room, 3 min lung washout (filtered mouthpiece) | NA | >12h | >12h | recorded | NA | NA | Recorded | >4 d | After | Before chemo and other cancer therapies | NA |
| Van de Goor et al.^52^, 2016 | AENOSE filter | Recorded (def as no food >4h before session) | Recorded (def as no drink >4h before session, except non cloric liquid 2 h before session) | NA | Recorded (def as smoking in previos month) | NA | NA | Recorded | After | After | Prior | NA |
| Wang et al.^53^, 2014 | Parallel collection of ambient air | Within 24 h after overnight fasting | Within 24 h after overnight fasting | NA | NA | NA | NA | See eligibility criteria | After | After | Before | NA |

**Supplement Table 3. Overview of identified VOCs**

|  | Gastro-esophageal cancer studies (n=8) | | | | | | | | Pancreatic cancer studies (n=2) | | Colorectal cancer studies (n=5) | | | | |  |
| --- | --- | --- | --- | --- | --- | --- | --- | --- | --- | --- | --- | --- | --- | --- | --- | --- |
| Volatile Organic Compounds that were found to be significant at alpha <0.05 | **AMAL et al.^32^, 2016** | **CHEN et al.^33^, 2016** | **KUMAR et al.^42^, 2013** | **KUMAR et al.^41^, 2015** | **MARKAR et al.^43^, 2018** | **SHEHADA et al.^37^, 2015** | **TONG et al.^38^, 2017** | **XU et al.^39^, 2013** | **MARKAR et al.^46^, 2018** | **PRINCIVALLE et al.^47^, 2018** | **ALTOMARE et al.^49^, 2013** | **ALTOMARE et al.^48^, 2015** | **AMAL et al.^50^, 2016** | **PENG et al.^51^, 2010** | **WANG et al.^53^, 2014** | **Total** |
| 1. Decanal |  |  |  | x | x |  |  |  |  |  | x | x |  |  |  | 4 |
| 1. 1,3-dimethylbenzene |  |  |  |  |  |  |  |  |  |  | x | x |  | x |  | 3 |
| 1. Nonanal |  |  |  | x |  |  | x (o) |  |  |  | x | x |  |  |  | 4 |
| 1. 3-methylpentane |  | x |  |  |  |  |  |  |  |  | x | x |  |  |  | 3 |
| 1. 2-methylpentane |  | x |  |  |  |  |  |  |  |  | x | x |  |  |  | 3 |
| 1. Methylcyclopentane |  |  |  |  |  |  |  |  |  |  | x | x |  |  |  | 2 |
| 1. Methylcyclohexane |  |  |  |  |  |  |  |  |  |  | x | x |  |  |  | 2 |
| 1. Cyclohexane |  |  |  |  |  |  |  |  |  |  | x | x |  |  |  | 2 |
| 1. 4-methyl-2-pentanone |  |  |  |  |  |  |  |  |  |  | x | x |  |  |  | 2 |
| 1. 1,4 dimethylbenzene |  |  |  |  |  |  |  |  |  |  | x | x |  |  |  | 2 |
| 1. 1,2-pentadiene |  |  |  |  |  |  |  |  |  |  | x | x |  |  |  | 2 |
| 1. 4-methyloctane | O (l) |  |  |  |  |  |  |  |  |  | x |  | x |  |  | 3 |
| 1. Undecane |  |  |  |  |  |  |  |  | x |  |  | x |  |  |  | 2 |
| 1. Dodecane |  | x |  |  |  |  |  |  |  |  |  | x |  |  |  | 2 |
| 1. 1,2,3-Trimethylbenzene | x (m) |  |  |  |  |  |  |  |  |  |  | x |  |  |  | 2 |
| 1. Acetone |  | x |  | O (e) |  |  |  |  | x |  |  |  | x |  |  | 4 |
| 1. Tetradecane |  | x |  |  |  |  |  |  | x |  |  |  |  |  |  | 2 |
| 1. Pentane |  |  | x |  |  |  |  |  | x |  |  |  |  |  |  | 2 |
| 1. Hexane |  | x |  |  |  |  |  |  | x |  |  |  |  |  |  | 2 |
| 1. Ammonia |  |  |  | O (f) |  |  |  |  |  | x |  |  |  |  |  | 2 |
| 1. Furfural (Furfuraldehyde) | x (i) |  |  |  |  | x |  | x |  |  |  |  |  |  |  | 3 |
| 1. Hexadecane | x (k) |  |  |  |  |  | x (n) |  |  |  |  |  |  |  |  | 2 |
| 1. 2-Butoxy-ethanol | x (j) |  |  |  |  |  |  | x |  |  |  |  |  |  |  | 2 |
| 1. 2-Propenenitril | x (h) |  |  |  |  | x |  | x |  |  |  |  |  |  |  | 3 |
| 1. Isoprene |  | x |  | O (g) |  |  |  | x |  |  |  |  |  |  |  | 3 |
| 1. Methanol |  |  | x (b) | O (d) |  |  |  |  |  |  |  |  |  |  |  | 2 |
| 1. Ethyl phenol |  |  | x (a) | x |  |  |  |  |  |  |  |  |  |  |  | 2 |
| 1. Butyric acid |  |  |  | x (c) | x |  |  |  |  |  |  |  |  |  |  | 2 |
| 1. Pentaioic acid |  |  |  | x | x |  |  |  |  |  |  |  |  |  |  | 2 |
| 1. Hexanoic acid |  |  |  | x | x |  |  |  |  |  |  |  |  |  |  | 2 |
| 1. Butanal |  |  |  | x | x |  |  |  |  |  |  |  |  |  |  | 2 |
| 1. 6-Methyl-5-hepten-2-one |  |  |  |  |  | x |  | x |  |  |  |  |  |  |  | 2 |

**Additional information on the table:**

X = Significantly present in all subgroup analyses (p<0.05); X ( ) = Significantly present in at least one or more subgroup analyses, see below for additional information; O = Not significantly present but mentioned in article

***Gastro esophageal cancer studies:***

a. Cancer vs. positive controls p=0.044, Cancer vs. healthy controls p= not significant.

b. Cancer vs. positive controls p=not sig., Cancer vs. healthy controls p=0.007

c. EC vs. non-cancer p=0.007, GC vs. non cancer p=0.1, AC vs. non-cancer p=0.005

d. EC vs. non-cancer p=0.87, GC vs. non cancer p=0.65, AC vs. non-cancer p=0.89

e. EC vs. non-cancer p=0.17, GC vs. non cancer p=0.17, AC vs. non-cancer p=0.09

f. EC vs. non-cancer p=0.21, GC vs. non cancer p=0.11, AC vs. non-cancer p=0.11

g. EC vs. non-cancer p=0.18, GC vs. non cancer p=0.38, AC vs. non-cancer p=0.14

h. GC vs. OLGIM 0-IV p<0.0001, GC vs. OLGIM 0 p= 0.0001, GC vs. OLGIM I-IV p= 0.0001

i. GC vs. OLGIM 0-IV p= 0.0001,

j. GC vs. OLGIM 0-IV p=0.0002, GC vs. OLGIM 0-II p=0.019

k. GC vs. OLGIM o-IV p<0.0001, GC vs. OLGIM 0 p=0.0001, GC vs. OLGIM 0-II p<0.0001, GC vs. OLGIM I-II p<0.0001, GC vs. OLGIM III-IV p<0.0001, GC vs. OLGIM I-IV p= 0.0004

l. Not significant for GC

m. GC vs. OLGIM I-IV p=0.0002

n. Carcinoma vs. normal p=0.000104, Carcinoma vs. gastric ulcer p=not significant, Carcinoma vs. Gastritis p=not significant

o. Carcinoma vs. normal p=not significant, Carcinoma vs. Gastric ulcer p= not significant, Carcinoma vs. Gastritis p<0.0001

**Supplement Table 4: Overview of all VOCs that were differently present between cancer patients and the control group.**

|  | **Gastro-esophageal cancer studies**  **(n=9)** | | | | | | | | | **Liver cancer studies (n=1)** | **Pancreas cancer studies**  **(n=2)** | | **Colorectal cancer studies (n=5)** | | | | |
| --- | --- | --- | --- | --- | --- | --- | --- | --- | --- | --- | --- | --- | --- | --- | --- | --- | --- |
| **Volatile Organic Compounds that were found to be significant at alpha <0.05** | **AMAL et al.^32^, 2016** | **CHEN et al al.^33^, 2016** | **KUMAR et al.^42^, 2013** | **KUMAR et al.^41^, 2015** | **MARKAR et al.^43^, 2018** | **SHEHADA et al.^37^, 2015** | **TONG et al.^38^, 2017** | **XU et al.^39^, 2013** | **ZOU et al.^44^, 2016** | **QIN et al.^45^, 2010** | **MARKAR et al.^46^, 2018** | **PRINCIVALLE et al.^47^, 2018** | **ALTOMARE et al.^31^, 2013** | **ALTOMARE et al.^48^, 2015** | **AMAL et al.^50^, 2016** | **PENG et al.^51^, 2010** | **WANG et al.^53^, 2014** |
| 1. 1.3-dimethyl benzene |  |  |  |  |  |  |  |  |  |  |  |  | X | X |  | X |  |
| 1. 1 iodo nonane |  |  |  |  |  |  |  |  |  |  |  |  |  |  |  | X |  |
| 1. 1.1-(butenylidene)bis benzene |  |  |  |  |  |  |  |  |  |  |  |  |  |  |  | X |  |
| 1. 1.1-dimethylethyl)thio aceticacid |  |  |  |  |  |  |  |  |  |  |  |  |  |  |  | X |  |
| 1. 2 amino 5 isopropyl-8-methyl-1öazulenecarbonitrile |  |  |  |  |  |  |  |  |  |  |  |  |  |  |  | X |  |
| 1. 4-(4-popylcylohexyl)-4-cyano 1.1-biphenyl-4-yl-ester benzoic acid |  |  |  |  |  |  |  |  |  |  |  |  |  |  |  | X |  |
| 1. (4 methyl undecane) |  |  |  |  |  |  |  |  |  |  |  |  | X |  |  |  |  |
| 1. (Trimethylbenzene) |  |  |  |  |  |  |  |  |  |  |  |  | X |  |  |  |  |
| 1. 1,2-pentadiene |  |  |  |  |  |  |  |  |  |  |  |  | X | X |  |  |  |
| 1. 1,4 dimethylbenzene |  |  |  |  |  |  |  |  |  |  |  |  | X | X |  |  |  |
| 1. 2-Methylbutane |  |  |  |  |  |  |  |  |  |  |  |  | X | X |  |  |  |
| 1. 2-methylpentane |  | X |  |  |  |  |  |  |  |  |  |  | X | X |  |  |  |
| 1. 3-methylpentane |  | X |  |  |  |  |  |  |  |  |  |  | X | X |  |  |  |
| 1. 4-methyl-2-pentanone |  |  |  |  |  |  |  |  |  |  |  |  | X | X |  |  |  |
| 1. 4-methyloctane | O (l) |  |  |  |  |  |  |  |  |  |  |  | X | X | X |  |  |
| 1. Cyclohexane |  |  |  |  |  |  |  |  |  |  |  |  | X | X |  |  |  |
| 1. Decanal |  |  |  | X | X |  |  |  |  |  |  |  | X | X |  |  |  |
| 1. Methyl cyclopentane |  |  |  |  |  |  |  |  |  |  |  |  | X | X |  |  |  |
| 1. Methylcyclohexane |  |  |  |  |  |  |  |  |  |  |  |  | X | X |  |  |  |
| 1. Nonanal |  |  |  | X |  |  | X (za) |  |  |  |  |  | X | X |  |  |  |
| 1. 2,21-Dimethyldecane |  |  |  |  |  |  |  |  |  |  |  |  |  |  |  |  | X |
| 1. 21-Methylprpanoate |  |  |  |  |  |  |  |  |  |  |  |  |  |  |  |  | X |
| 1. 4 1-Ethyl-111-octyn1-31-o1 |  |  |  |  |  |  |  |  |  |  |  |  |  |  |  |  | X |
| 1. 61-t1-Butyl12,2,9,91-tetramethyl-13,51-decadien-171-yne |  |  |  |  |  |  |  |  |  |  |  |  |  |  |  |  | X |
| 1. Cyclohexanone |  |  |  |  |  |  |  |  |  |  |  |  |  |  |  |  | X |
| 1. Cyclooctylmethanol, |  |  |  |  |  |  |  |  |  |  |  |  |  |  |  |  | X |
| 1. Dodecanane 31-hydroxy-12,4,4 1-trimethylpenthyl |  |  |  |  |  |  |  |  |  |  |  |  |  |  |  |  | X |
| 1. Ethyl aniline |  |  |  |  |  |  |  |  |  |  |  |  |  |  |  |  | X |
| 1. Trans-121-Dodecen-111-o1 |  |  |  |  |  |  |  |  |  |  |  |  |  |  |  |  | X |
| 1. Acetone |  | X |  | O (e) |  |  |  |  |  |  | X |  |  |  | X |  |  |
| 1. Ethanol |  |  |  |  |  |  |  |  |  |  |  |  |  |  | X |  |  |
| 1. Ethyl acetate |  |  |  |  |  |  |  |  |  |  |  |  |  |  | X |  |  |
| 1. Decane |  |  |  |  |  |  |  |  |  | X (xyz) |  |  |  |  |  |  |  |
| 1. 3-hydroxy-2-butanone |  |  |  |  |  |  |  |  |  | X |  |  |  |  |  |  |  |
| 1. Styrene |  |  |  |  |  |  |  |  |  | X |  |  |  |  |  |  |  |
| 1. Ammonia |  |  |  | O (f) |  |  |  |  |  |  |  | X |  |  |  |  |  |
| 1. M112 |  |  |  |  |  |  |  |  |  |  |  | X |  |  |  |  |  |
| 1. M34 |  |  |  |  |  |  |  |  |  |  |  | X |  |  |  |  |  |
| 1. M43 |  |  |  |  |  |  |  |  |  |  |  | X |  |  |  |  |  |
| 1. M44 |  |  |  |  |  |  |  |  |  |  |  | X |  |  |  |  |  |
| 1. M62 |  |  |  |  |  |  |  |  |  |  |  | X |  |  |  |  |  |
| 1. M71 |  |  |  |  |  |  |  |  |  |  |  | X |  |  |  |  |  |
| 1. M74 |  |  |  |  |  |  |  |  |  |  |  | X |  |  |  |  |  |
| 1. M89 |  |  |  |  |  |  |  |  |  |  |  | X |  |  |  |  |  |
| 1. Sulfur oxide |  |  |  |  |  |  |  |  |  |  |  | X |  |  |  |  |  |
| 1. 1-Butanol |  |  |  |  |  |  |  |  |  |  | X (xyb) |  |  |  |  |  |  |
| 1. Amylene hydrate |  |  |  |  |  |  |  |  |  |  | X (xya) |  |  |  |  |  |  |
| 1. 1-(Methylthio)-propane |  |  |  |  |  |  |  |  |  |  | X |  |  |  |  |  |  |
| 1. Acetoin |  |  |  |  |  |  |  |  |  |  | X |  |  |  |  |  |  |
| 1. Benzaldehyde |  |  |  |  |  |  |  |  |  |  | X |  |  |  |  |  |  |
| 1. Formaldehyde |  |  |  |  |  |  |  |  |  |  | X |  |  |  |  |  |  |
| 1. Isopropylalcohol |  |  |  |  |  |  |  |  |  |  | X |  |  |  |  |  |  |
| 1. N-Hexane |  |  |  |  |  |  |  |  |  |  | X |  |  |  |  |  |  |
| 1. Pentane |  |  | X |  |  |  |  |  |  |  | X |  |  |  |  |  |  |
| 1. Tetradecane |  | X |  |  |  |  |  |  |  |  | X |  |  |  |  |  |  |
| 1. Undecane |  |  |  |  |  |  |  |  |  |  | X |  |  |  |  |  |  |
| 1. Butanal |  |  |  | X | X |  |  |  |  |  |  |  |  |  |  |  |  |
| 1. Butyric acid |  |  |  | X (c) | X |  |  |  |  |  |  |  |  |  |  |  |  |
| 1. Hexanoic acid |  |  |  | X | X |  |  |  |  |  |  |  |  |  |  |  |  |
| 1. Pentaioic acid |  |  |  | X | X |  |  |  |  |  |  |  |  |  |  |  |  |
| 1. Isoprene |  | X |  | O (g) |  |  |  | X |  |  |  |  |  |  |  |  |  |
| 1. 2-Butoxy-ethanol | X (j) |  |  |  |  |  |  | X |  |  |  |  |  |  |  |  |  |
| 1. 2-Propenenitril | X (h) |  |  |  |  | X |  | X |  |  |  |  |  |  |  |  |  |
| 1. 6-Methyl-5-hepten-2-one |  |  |  |  |  | X |  | X |  |  |  |  |  |  |  |  |  |
| 1. Furfural (Furfuraldehyde) | X (j) |  |  |  |  | X |  | X |  |  |  |  |  |  |  |  |  |
| 1. Methanol |  |  | X (b) | O (d) |  |  |  |  |  |  |  |  |  |  |  |  |  |
| 1. Ethyl phenol |  |  | X (a) | X |  |  |  |  |  |  |  |  |  |  |  |  |  |
| 1. Methyl phenol |  |  | X |  |  |  |  |  |  |  |  |  |  |  |  |  |  |
| 1. Heptanal |  |  |  | X |  |  |  |  |  |  |  |  |  |  |  |  |  |
| 1. Hexanal |  |  |  | X |  |  |  |  |  |  |  |  |  |  |  |  |  |
| 1. Methylphenol |  |  |  | X |  |  |  |  |  |  |  |  |  |  |  |  |  |
| 1. Octanal |  |  |  | X |  |  |  |  |  |  |  |  |  |  |  |  |  |
| 1. Pentanal |  |  |  | X |  |  |  |  |  |  |  |  |  |  |  |  |  |
| 1. Phenol |  |  |  | X |  |  |  |  |  |  |  |  |  |  |  |  |  |
| 1. 2-Butanone | X (o) |  |  |  |  |  |  |  |  |  |  |  |  |  |  |  |  |
| 1. Alpha-methyl-styrene | X (n) |  |  |  |  |  |  |  |  |  |  |  |  |  |  |  |  |
| 1. 1,2,3-Tri-methyl-benzene | X (m) |  |  |  |  |  |  |  |  |  |  |  |  |  |  |  |  |
| 1. Hexadecane | X (k) |  |  |  |  |  | X (r) |  |  |  |  |  |  |  |  |  |  |
| 1. Benzothiazole |  |  |  |  |  |  | X (zc) |  |  |  |  |  |  |  |  |  |  |
| 1. 5-Hepten-2-one, 6-methyl- |  |  |  |  |  |  | X (zb) |  |  |  |  |  |  |  |  |  |  |
| 1. 5,7-octadien-2-on,3-acetyl |  |  |  |  |  |  | X (z) |  |  |  |  |  |  |  |  |  |  |
| 1. Caprolactam |  |  |  |  |  |  | X (y) |  |  |  |  |  |  |  |  |  |  |
| 1. 1,6 Dioxacyclododecane-7,12-dione P |  |  |  |  |  |  | X (x) |  |  |  |  |  |  |  |  |  |  |
| 1. 3,5-Decadie—yn,6-t-buty-2,2,9,9-tetramethyl |  |  |  |  |  |  | X (w) |  |  |  |  |  |  |  |  |  |  |
| 1. 1,3-Dioxolane-2-methanol |  |  |  |  |  |  | X (v) |  |  |  |  |  |  |  |  |  |  |
| 1. Phosphonic acid |  |  |  |  |  |  | X (u) |  |  |  |  |  |  |  |  |  |  |
| 1. N,N,-Dimethylacetamide |  |  |  |  |  |  | X (t) |  |  |  |  |  |  |  |  |  |  |
| 1. Undecane, 3.8,dimethyl- |  |  |  |  |  |  | X (s) |  |  |  |  |  |  |  |  |  |  |
| 1. 1,3-Dioxolan-2-one |  |  |  |  |  |  | X (q) |  |  |  |  |  |  |  |  |  |  |
| 1. 2,2-Butanediol |  |  |  |  |  |  | X (p) |  |  |  |  |  |  |  |  |  |  |
| 1. 2-methylhexane |  | X |  |  |  |  |  |  |  |  |  |  |  |  |  |  |  |
| 1. 2,3-dimethylpentane |  | X |  |  |  |  |  |  |  |  |  |  |  |  |  |  |  |
| 1. 3-methylhexane |  | X |  |  |  |  |  |  |  |  |  |  |  |  |  |  |  |
| 1. Dodecane |  | X |  |  |  |  |  |  |  |  |  |  |  |  |  |  |  |
| 1. Hexane |  | X |  |  |  |  |  |  |  |  |  |  |  |  |  |  |  |
| 1. Hexanol |  | X |  |  |  |  |  |  |  |  |  |  |  |  |  |  |  |
| 1. Menthol |  | X |  |  |  |  |  |  |  |  |  |  |  |  |  |  |  |
| 1. Phenyl acetate |  | X |  |  |  |  |  |  |  |  |  |  |  |  |  |  |  |
| 1. Pivalic acid |  | X |  |  |  |  |  |  |  |  |  |  |  |  |  |  |  |
| 1. 1. m/z 136 |  |  |  |  |  |  |  |  | X |  |  |  |  |  |  |  |  |
| 1. 2. m/z 34 |  |  |  |  |  |  |  |  | X |  |  |  |  |  |  |  |  |
| 1. 3. m/z 63 |  |  |  |  |  |  |  |  | X |  |  |  |  |  |  |  |  |
| 1. 4. m/z 27 |  |  |  |  |  |  |  |  | X |  |  |  |  |  |  |  |  |
| 1. 5. m/z 95 |  |  |  |  |  |  |  |  | X |  |  |  |  |  |  |  |  |
| 1. 6. m/z 107 |  |  |  |  |  |  |  |  | X |  |  |  |  |  |  |  |  |
| 1. 7. m/z 45 |  |  |  |  |  |  |  |  | X |  |  |  |  |  |  |  |  |
| **Additional information on the table:**  X = Significantly expressed in all subgroup analyses; X ( ) = Significantly expressed in at least one or more subgroup analyses, see below for additional information; O = Not significantly expressed but mentioned in article  ***Pancreas cancer studies:***  Xya. Cancer vs. no-cancer p=0.457, Adenocarcinoma vs. cancer p <0.001, (less in cancer)  Xyb. Cancer vs. no-cancer p=0.407, Adenocarcinoma vs. cancer p = 0.005, (less in cancer)  ***Liver cancer studies:***  Xyz. HV vs HCC p <0.001, HC vs HCC p= 0.076  ***Gastro esophageal cancer studies:***  a. Cancer vs. positive controls p=0.044, Cancer vs. healthy controls p= not significant.  b. Cancer vs. positive controls p=not sig., Cancer vs. healthy controls p=0.007  c. EC vs. non-cancer p=0.007, GC vs. non cancer p=0.1, AC vs. non-cancer p=0.005  d. EC vs. non-cancer p=0.87, GC vs. non cancer p=0.65, AC vs. non-cancer p=0.89  e. EC vs. non-cancer p=0.17, GC vs. non cancer p=0.17, AC vs. non-cancer p=0.09  f. EC vs. non-cancer p=0.21, GC vs. non cancer p=0.11, AC vs. non-cancer p=0.11  g. EC vs. non-cancer p=0.18, GC vs. non cancer p=0.38, AC vs. non-cancer p=0.14  h. GC vs OLGIM 0-IV p<0.0001, GC vs OLGIM 0 p= 0.0001, GC vs OLGIM I-IV p= 0.0001  i. GC vs OLGIM 0-IV p= 0.0001, j. GC vs OLGIM 0-IV P=0.0002, GC vs OLGIM 0-II p=0.019  k. GC vs OLGIM o-IV p<0.0001, GC vs OLGIM 0 p=0.0001, GC vs OLGIM 0-II p<0.0001, GC vs OLGIM I-II p<0.0001, GC vs OLGIM III-IV p<0.0001, GC vs OLGIM I-IV p= 0.0004  l. Not significant for GC  m. GC vs OLGIM I-IV p=0.0002  n. GC vs OLGIM 0 p<0.0001, GC vs OLGIM 0-II p<0.0001, GC vs OLGIM I-IV p<0.0001  o. GC vs OLGIM 0-II p=0.009  p. Carcinoma vs. normal p= 0.00249, Carcinoma vs. gastric ulcer p=not significant, Carcinoma vs. Gastritis p=not significant  q. Carcinoma vs. normal p=0.00256, Carcinoma vs. gastric ulcer p=not significant, Carcinoma vs. Gastritis p=not significant  r. Carcinoma vs. normal p=0.000104, Carcinoma vs. gastric ulcer p=not significant, Carcinoma vs. Gastritis p=not significant  s. Carcinoma vs. normal p=0.013, Carcinoma vs. gastric ulcer p=not significant, Carcinoma vs. Gastritis p=not significant  t. Carcinoma vs. normal p=not significant, Carcinoma vs. Gastric ulcer p= 0.000834, Carcinoma vs. Gastritis p<0.0001  u. Carcinoma vs. normal p=not significant, Carcinoma vs. Gastric ulcer p=0.000301, Carcinoma vs. Gastritis p<0.0001  v. Carcinoma vs. normal p=not significant, Carcinoma vs. Gastric ulcer p=0.000438, Carcinoma vs. Gastritis p=0.00201  w. Carcinoma vs. normal p=not significant, Carcinoma vs. Gastric ulcer p= not significant, Carcinoma vs. Gastritis p<0.001  x. Carcinoma vs. normal p=not significant, Carcinoma vs. Gastric ulcer p= not significant, Carcinoma vs. Gastritis p=<0.0001  y. Carcinoma vs. normal p=not significant, Carcinoma vs. Gastric ulcer p= not significant, Carcinoma vs. Gastritis p<0.0001  z. Carcinoma vs. normal p=not significant, Carcinoma vs. Gastric ulcer p= not significant, Carcinoma vs. Gastritis p<0.0001  Za. Carcinoma vs. normal p=not significant, Carcinoma vs. Gastric ulcer p= not significant, Carcinoma vs. Gastritis p<0.0001  Zb. Carcinoma vs. normal p=not significant, Carcinoma vs. Gastric ulcer p= not significant, Carcinoma vs. Gastritis p<0.0001  Zc. Carcinoma vs. normal p=not significant, Carcinoma vs. Gastric ulcer p= not significant, Carcinoma vs. Gastritis px=0.00942 | | | | | | | | | | | | | | | | | |
